# DIFFERENT INTERPRETATIONS OF “HONOR YOUR PARENTS”: IMPLICATIONS FOR OBLIGATION OF PARENTAL CAREGIVING

**DOI:** 10.1093/geroni/igae098.1352

**Published:** 2024-12-31

**Authors:** Darlingtina Esiaka, Elizabeth Luth

**Affiliations:** University of Kentucky College of Medicine, University of Kentucky, Kentucky, United States; Rutgers University, New Brunswick, New Jersey, United States

## Abstract

Although the concept of “honor your parents” is [universally] important, its understanding and manifestation vary by cultural context and community. This variation potentially has important implications for how parental caregiving obligations are perceived and performed. The current study explored the diverse conceptualizations of “honoring your parents” in the United States, Ghana, and Nigeria. It also assessed how the interpretations influence individuals’ perceptions of caregiving obligations to older parents. Through semi-structured interviews with 153 participants, we found that in the United States, “honoring your parents” is predominantly viewed through the lens of emotional care rather than material care and instrumental support. In contrast, individuals in Nigeria and Ghana strongly associate the concept with providing tangible assistance and hands-on care to parents. Our findings shed light on cultural nuances and expectations regarding parental caregiving, offering insights into potential strategies to address the diverse caregiving needs of aging populations.

